# Oral Community Interactions of *Filifactor alocis In Vitro*

**DOI:** 10.1371/journal.pone.0076271

**Published:** 2013-10-03

**Authors:** Qian Wang, Christopher J. Wright, Huang Dingming, Silvia M. Uriarte, Richard J. Lamont

**Affiliations:** 1 Center for Oral Health and Systemic Disease, School of Dentistry, University of Louisville, Louisville, Kentucky, United States of America; 2 Department of Medicine, University of Louisville, Louisville, Kentucky, United States of America; 3 State Key Laboratory of Oral Diseases, Sichuan University, Chengdu, China; University of Oklahoma Health Sciences Center, United States of America

## Abstract

*Filifactor alocis* is a gram positive anaerobe that is emerging as an important periodontal pathogen. In the oral cavity *F. alocis* colonizes polymicrobial biofilm communities; however, little is known regarding the nature of the interactions between *F. alocis* and other oral biofilm bacteria. Here we investigate the community interactions of two strains of *F. alocis* with *Streptococcus gordonii*, *Fusobacterium nucleatum*, *Porphyromonas gingivalis* and *Aggregatibacter actinomycetemcomitans*, organisms with differing pathogenic potential in the oral cavity. In an in vitro community development model, *S. gordonii* was antagonistic to the accumulation of *F. alocis* into a dual species community. In contrast, *F. nucleatum* and the type strain of *F. alocis* formed a synergistic partnership. Accumulation of a low passage isolate of *F. alocis* was also enhanced by *F. nucleatum*. In three species communities of *S. gordonii*, *F. nucleatum* and *F. alocis*, the antagonistic effects of *S. gordonii* superseded the synergistic effects of *F. nucleatum* toward *F. alocis*. The interaction between *A. actinomycetemcomitans* and *F. alocis* was strain specific and *A. actinomycetemcomitans* could either stimulate *F. alocis* accumulation or have no effect depending on the strain. *P. gingivalis* and *F. alocis* formed heterotypic communities with the amount of *P. gingivalis* greater than in the absence of *F. alocis*. However, while *P. gingivalis* benefited from the relationship, levels of *F. alocis* in the dual species community were lower compared to *F. alocis* alone. The inhibitory effect of *P. gingivalis* toward *F. alocis* was dependent, at least partially, on the presence of the Mfa1 fimbrial subunit. In addition, AI-2 production by *P. gingivalis* helped maintain levels of *F. alocis*. Collectively, these results show that the pattern of *F. alocis* colonization will be dictated by the spatial composition of microbial microenvironments, and that the organism may preferentially accumulate at sites rich in *F. nucleatum.*

## Introduction

The dental plaque biofilm is comprised of complex communities of microorganisms embedded on tooth surfaces, and is a direct precursor of periodontal disease [1]–[3]. Until fairly recently, a limited number of organisms in the subgingival biofilm, the so called ‘red complex’, were considered the predominant pathogens in chronic and severe cases of adult periodontitis [4], [5]. However, microbiome studies over the last several years have changed our understanding of the multispecies microbial communities that inhabit the oral cavity. The microbial composition of periodontal disease lesions is much more varied than previously recognized and contains high levels of fastidious and as yet-to-be-cultivated taxons [6]. Organisms such as *Selenomonas*, *Synergistes*, *Desulfobulbus*, TM7 and *Filifactor alocis* have been identified as potential pathogens in a number of independent studies [6]–[10].

*F. alocis* is a Gram-positive, slow-growing, obligate anaerobic rod that is found at increased frequency and in higher numbers in periodontal disease sites compared with healthy sites [6], [8], [9], [11]–[13]. In addition, *F. alocis* is emerging as an important organism in aggressive periodontitis in children [14], endodontic lesions [15] and pericoronitis [16]. Study of the pathogenic properties of *F. alocis* is now important to impute a causal association between *F. alocis* and periodontal disease. In that regard, *F. alocis* has a number of characteristics consistent with that of a periodontal pathogen. The organism is resistant to oxidative stress and generally proinflammatory and proapoptotic [17], [18]. Furthermore, *F. alocis* produces several proteases and neutrophil-activating protein A which are upregulated during internalization within epithelial cells [19].

An important early step in the colonization process of periodontal pathogens is the ability to adhere to oral surfaces and accumulate in physiologically compatible heterotypic communities. Schlafer et al. [20] examined the topology of *F. alocis* within *in vivo* grown subgingival biofilms from periodontitis patients. *F. alocis* was frequently present in densely packed groups as a part of concentric bacterial aggregates, and in mushroom-like protuberances on the surface of the biofilm. *F. alocis* also formed structures resembling test-tube brushes (often observed in dental biofilms [21]). It is likely, therefore, that *F. alocis* can interact with a variety of oral bacteria and participate in community development. In this study we utilize *in vitro* models to examine the community forming interactions of *F. alocis* with common oral organisms of varying degrees of pathogenicity.

## Materials and Methods

### Ethics Statement

Saliva collection was approved by the University of Louisville IRB, Protocol # 12.0345 and designated as non-human subjects research as saliva was collected from study principal investigator only.

### Bacteria and Culture Conditions

*Filifactor alocis* strain ATCC 38596 and low passage clinical isolate D-62D were cultured in *F. alocis* broth (FAB) comprised of Brain Heart Infusion broth (BHI) supplemented with yeast extract (0.5 mg/ml), L-cysteine (50 μg/ml), and 20% arginine [17]. *P. gingivalis* ATCC 33277, isogenic mutants Δ*luxS* and Δ*mfa*1 and complemented *mfa1* mutant, CΔ*mfa*1 [22] were cultured in trypticase soy broth (TSB) supplemented with yeast extract (1 mg/ml), hemin (5 μg/ml) and menadione (1 μg/ml). *Fusobacterium nucleatum* ATCC 25586 was cultured in BHI supplemented with hemin (5 μg/ml) and menadione (1 μg/ml). *Aggregatibacter actinomycetemcomitans* strain 652 was grown in BHI, and *Streptococcus gordonii* strain DL1 was grown in Todd-Hewitt broth. *F. alocis*, *P. gingivalis*, *F. nucleatum* and *S. gordonii* were cultured anaerobically. *A. actinomycetemcomitans* was cultured under microaerophilic conditions. All organisms were grown at 37°C.

### Saliva Collection

Whole saliva was collected from a healthy volunteer, and dithiothreitol was added to a final concentration of 2.5 mM. Particulate matter was removed by centrifugation at 10 000 *g* for 10 min. Clarified saliva was diluted to 10% with distilled water, filtered through 0.2 µm pore size nitrocellulose and stored at −80°C. Glass coverslips were reacted with 0.5 ml of 10% saliva (4°C for 16 h) and rinsed with PBS prior to use.

### Community Analysis by Confocal Laser Scanning Microscopy (CLSM)

Quantitative and structural analysis of homotypic and heterotypic communities was accomplished by CLSM and subsequent image analysis essentially as previously described [23]. A) Single species. *S. gordonii*, *F. nucleatum*, *A. actinomycetemcomitans* or *P. gingivalis* cells (2×10^8^) were stained with hexidium iodide (15 μg/ml; Invitrogen, Carlsbad, CA), and *F. alocis* cells (2×10^8^) were stained with fluorescein isothiocyanate (FITC, 4 µg/ml, Invitrogen). Bacteria were cultured in individual chambers of a Culture Well chambered coverglass system (Grace Bio Laboratories, Bend, OR) in FAB (unless otherwise stated) anaerobically with rocking at 37°C. B) Dual species. *S. gordonii, F. nucleatum*, *A. actinomycetemcomitans* or *P. gingivalis* cells (2×10^8^ unless otherwise stated) were stained with hexidium iodide were cultured anaerobically in FAB on coverslips overnight with rocking at 37°C. *F. alocis* cells (5×10^7^) were stained with FITC and reacted with the partner species anaerobically with rocking in FAB at 37°C. C) Three species. *S. gordonii* stained with hexidium iodide, and *F. nucleatum* stained with 4′,6-diamino-2-phenylindole (DAPI, 1 μg/ml; Invitrogen) were co-cultured on coverslips overnight in FAB anaerobically with rocking at 37°C. After washing, FITC-labelled *F. alocis* were reacted with the dual species substratum anaerobically with rocking in FAB at 37°C. Coverslips with assembled communities were washed, and quantitative and structural analysis was performed on an Olympus confocal laser scanning microscope (FV1000) with a ×60 objective. A series of 0.5-µm-deep optical fluorescent *x–y* sections (120×120 µm) were collected to create digitally reconstructed 3D images with Volocity software (Perkin Elmer, Waltham, MA).

### Statistical Analysis

Community assays were repeated independently four times in triplicate and analysed with a Student’s unpaired two-tailed t-test. Pearson’s correlation coefficient (PCC) in Volocity was used to ascertain the degree of inter-species colocalization [24], [25].

## Results

### Monospecies Communities

**Figure 1:**
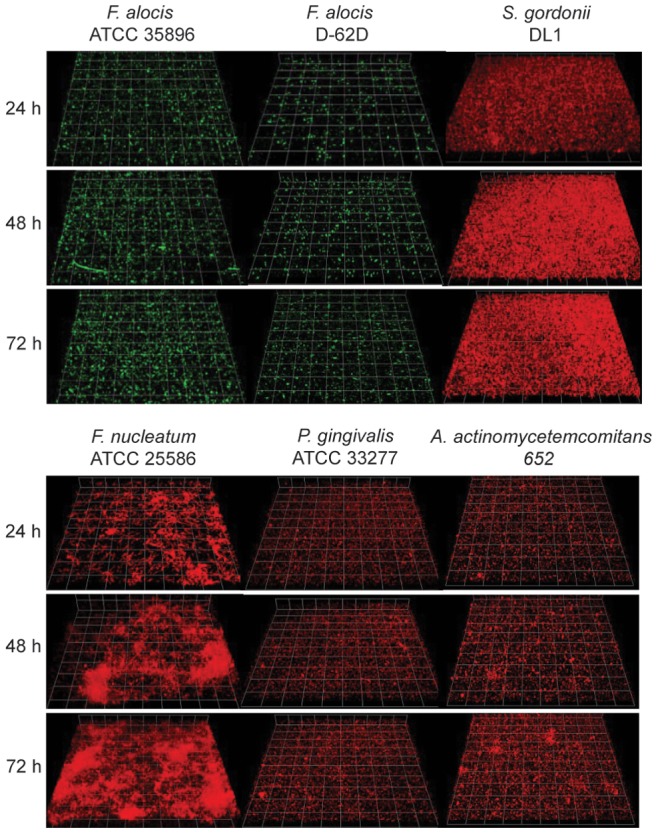
CLSM projections of monospecies communities of *F.alocis* strains ATCC 35896 and D-62D (green, stained with FITC), *S. gordonii* DL-1, *F. nucleatum* ATCC25586, *A. actinomycetemcomitans* 652, or *P. gingivalis* ATCC33277 (red, stained with hexidium iodide) after 24 h, 48 h, and 72 h.

Initially, the structural and quantitative properties of single species communities were determined at 24, 48 and 72 h (Fig. 1). In monospecies accumulations, *F. alocis, A. actinomycetemcomitans* and *P. gingivalis* sporadically formed small microcolonies. Community formation by the *F. alocis* low passage clinical isolate D-62D was sparser compared to the type strain at all time points. *F. nucleatum* communities developed in unevenly distributed dense clusters. *In vivo S. gordonii* attaches to the salivary pellicle on enamel surfaces [3], and hence a saliva-coated glass surface was used for *S. gordonii* community formation. While processing of saliva by centrifugation and filtering can remove mucins and anti-microbial compounds which could influence bacterial growth, *S. gordonii* developed a markedly thick biofilm, up to 10 µm deep. The biomass of all of the species tested increased over time.

### Dual Species Communities

#### A) *F. alocis-S. gordonii*

**Figure 2:**
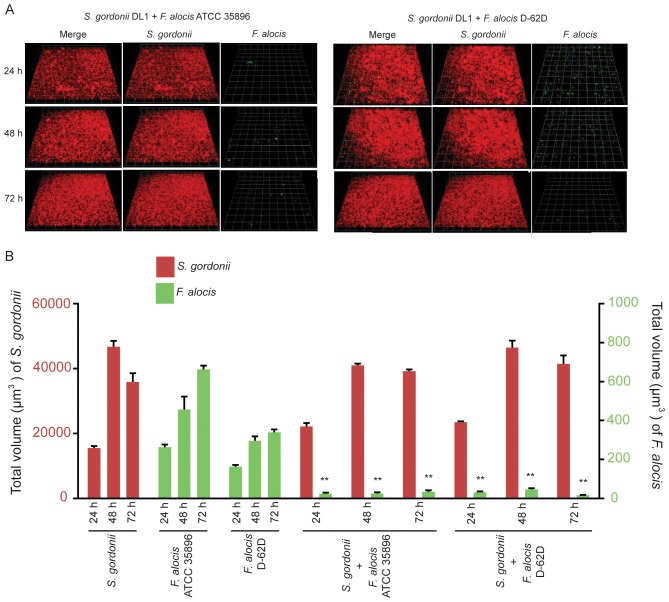
Dual-species community formation between *F. alocis* and *S. gordonii* analyzed by CLSM. A. *S. gordonii* DL-1 (red, stained with hexidium iodide) was cultured on a saliva-coated coverglass. *F. alocis* strains ATCC 35896 (upper left panel) and D-62D (upper right panel) were stained with FITC (green) and reacted with *S. gordonii* for 24 h, 48 h and 72 h. B. Time-resolved changes in the biovolume of *S. gordonii* DL-1, *F.alocis* ATCC 35896 and D-62D in dual species communities. Data are representative of four independent replicates. P-value compared with control single species communities was calculated by t-test, and significant differences are at p<0.001(**).

The ability of *F. alocis* to accumulate on substrata of *S. gordonii* attached to saliva-coated glass coverslips was investigated. Both *F. alocis* ATCC 35896 and D-62D strains exhibited sparse accumulation with *S. gordonii* DL1 (Fig. 2A). Quantitative measurement of the dual-species communities (Fig. 2B) demonstrated that *S. gordonii* did not show a significant difference compared to accumulation in single species communities. However, the accumulation of *F. alocis* strains with *S. gordonii* showed a dramatic decrease compared to *F. alocis* alone. At 72 h, the biovolume of strain ATCC 35896 accumulation was reduced 19-fold (p<0.001) by *S. gordonii* whereas D-62D accumulation was reduced 21-fold (p<0.001). This result suggests that the presence of *S. gordonii* is strongly inhibitory to *F. alocis* in *F. alocis*-*S. gordonii* heterotypic communities.

#### B) *F. alocis-F. nucleatum*

**Figure 3:**
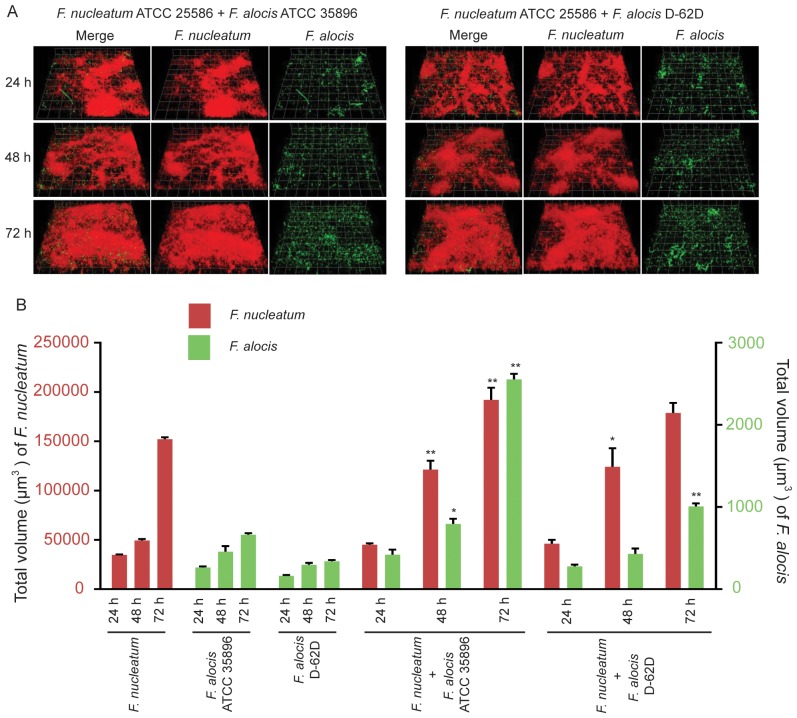
Dual-species community formation between *F. alocis* and *F. nucleatum* analyzed by CLSM. A. *F. nucleatum* ATCC 25586 (red, stained with hexidium iodide) was cultured on glass coverslips. *F. alocis* strains ATCC 35896 (upper left panel) and D-62D (upper right panel) were stained with FITC (green) and reacted with *F. nucleatum* for 24 h, 48 h and 72 h. B. Time-resolved changes in the biovolume of *F. nucleatum* ATCC 25586, *F. alocis* ATCC 35896 and D-62D in dual species communities. Data are representative of four independent replicates. P-value compared with control single species communities was calculated by t-test, and significant differences are at p<0.05 (*) or p<0.01(**).

Fig. 3A shows that both *F. alocis* strains accumulated around regions of *F. nucleatum* abundance. Time-resolved inspection of dual-species biofilm development (Fig. 3B) revealed that *F. alocis* strains exhibited an increase in total biovolume: after 48 h in the case of strain ATCC 35896, and after 72 h with strain D-62D. This was accompanied by mutualistic growth of *F. nucleatum* after 48 h, although synergism was lost at 72 h with strain D-62D. Collectively, these results indicate that *F. nucleatum* and *F. alocis* can exhibit a synergistic relationship in the accumulation of dual-species biofilms.

#### C) *F. alocis-A. actinomycetemcomitans*

**Figure 4:**
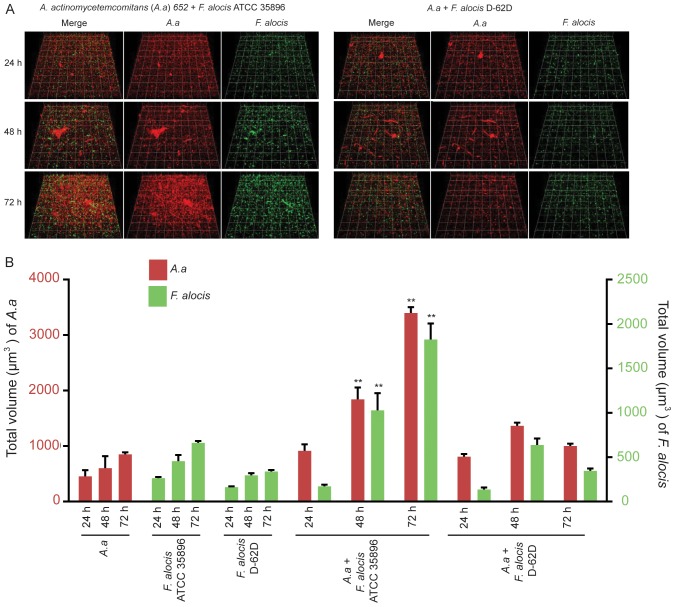
Dual-species community formation between *F. alocis* and *A. actinomycetemcomitans* analyzed by CLSM. A. *A. actinomycetemcomitans* 652 (red, stained with hexidium iodide) was cultured on glass coverslips. *F. alocis* strains ATCC 35896 (upper left panel) and D-62D (upper right panel) were stained with FITC (green) and reacted with *A. actinomycetemcomitans* for 24 h, 48 h and 72 h. B. Time-resolved changes in the biovolume of *A. actinomycetemcomitans* 652, *F. alocis* ATCC 35896 and D-62D in dual species communities. Data are representative of four independent replicates. P-value compared with control single species communities was calculated by t-test, and significant differences are at p<0.01(**).

Large aggregations of *A. actinomycetemcomitans* formed between 48 and 72 h of co-culture (Fig. 4A). The biovolume of both *F. alocis* ATCC 35896 and *A. actinomycetemcomitans* in heterotypic communities increased following 48 h incubation indicating mutualistic growth. In contrast, co-culture of *F. alocis* strain D-62D with *A. actinomycetemcomitans* did not stimulate the accumulation of either species, indicating strain-specific *F. alocis* interactions with *A. actinomycetemcomitans* (Fig. 4B).

#### D) *F. alocis-P. gingivalis*

**Figure 5:**
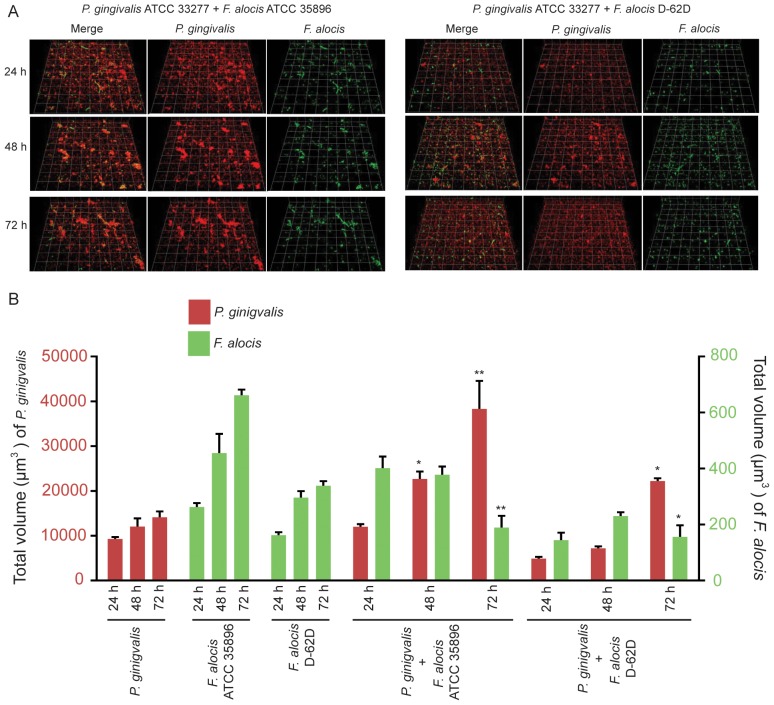
Dual-species community formation between *F. alocis* and *P. gingivalis* analyzed by CLSM. A. *P. gingivalis* ATCC 33277 (red, stained with hexidium iodide) was cultured on glass coverslips. *F. alocis* strains ATCC 35896 (upper left panel) and D-62D (upper right panel) were stained with FITC (green) and reacted with *P. gingivalis* for 24 h, 48 h and 72 h. B. Time-resolved changes in the biovolume of *P. gingivalis* ATCC 33277, *F. alocis* ATCC 35896 and D-62D in dual species communities. Data are representative of four independent replicates. P-value compared with control single species communities was calculated by t-test, and significant differences are at p<0.05 (*) or p<0.01(**).

Heterotypic *F. alocis*–*P. gingivalis* communities are shown in Fig. 5A. On substrata of *P. gingivalis,* both *F. alocis* strains showed accumulation over a 72 h period; however, the biovolume of *F. alocis* was reduced with *P. gingivalis* as compared with *F. alocis* alone (Fig. 5B). In contrast, *P. gingivalis* was capable of growth in the presence of *F. alocis*, reaching greater biovolume at 72 h compared to *P. gingivalis* alone (Fig. 5B). These results reveal that *F. alocis* and *P. gingivalis* can assemble into heterotypic communities; however, while *P. gingivalis* benefits from this interaction, accumulation of *F. alocis* is inhibited.

### Colocalization Within Communities

**Figure 6:**
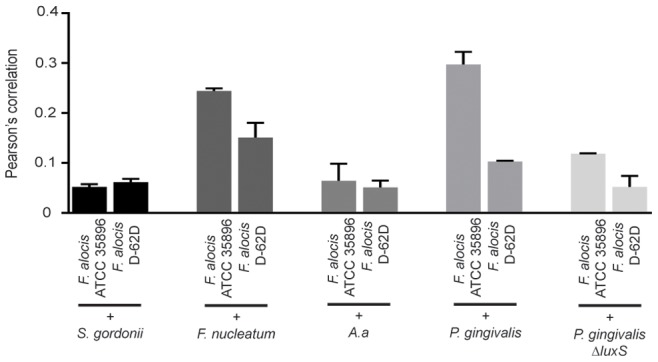
Colocalization of *F. alocis* with partner species in heterotypic communities. Pearson’s correlation was determined using Volocity software. Data are representative of four independent replicates.

To investigate initial physical interactions between bacteria in dual species communities, colocalization analysis with Volocity software was performed, employing Pearson’s Correlation Coefficient (PCC) (Fig. 6). *F. alocis*-*S. gordonii* heterotypic communities showed a low level of colocalization, reflective of the antagonistic relationship of *S. gordonii* toward *F. alocis*. In contrast, *F. alocis* and *F. nucleatum*, which exhibit synergy, displayed a higher degree of colocalization in communities. Colocalization between *A. actinomycetemcomitans* and both strains of *F. alocis* was low, and hence the mutualistic growth between *A. actinomycetemcomitans* and *F. alocis* ATCC 35896 may depend on soluble secreted factors. *F. alocis* colocalization with *P. gingivalis* was relatively high, indicating that the two species physically interact before the inhibitory effect of *P. gingivalis* is manifest.

### Interaction Between *P. gingivalis* and *F. alocis*

**Figure 7:**
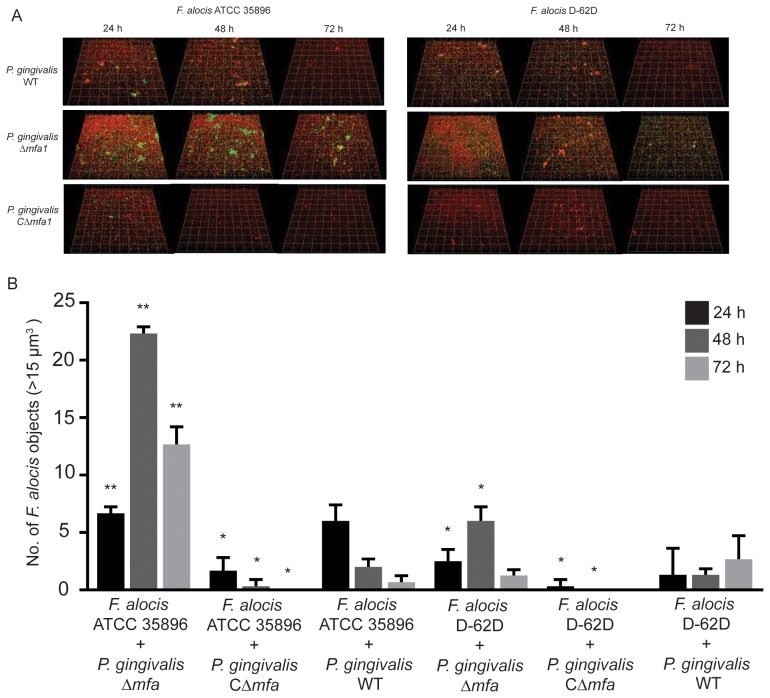
Dual-species community formation between *F. alocis* and *P. gingivalis mfa1* mutants analyzed by CLSM. A. *P. gingivalis* ATCC 33277 (WT), Δ*mfa1* and cΔ*mfa1* (1 × 10^8^, red, stained with hexidium iodide) were cultured on glass coverslips. *F. alocis* strains ATCC 35896 (upper left panel) and D-62D (upper right panel) were stained with FITC (green) and reacted with the *P. gingivalis* strains for 24 h, 48 h and 72 h. B. Quantification of accumulation of *P. gingivalis* objects greater than 15 um^3^ in dual species communities by Volocity software. Data are representative of four independent replicates. P-values at each time point was calculated by t-test, and significant differences from WT are at p<0.05 (*) or p<0.01(**).

Our data indicated that *F. alocis* and *P. gingivalis* physically interact and hence we utilized a panel of *P. gingivalis* mutants deficient in expression of major surface adhesins to begin to investigate the molecular basis of the interaction. Loss of the major (FimA) fimbriae or the internalin family protein InlJ had no effect on community formation with *F. alocis* (not shown). In contrast, loss of the minor fimbriae (Mfa1) increased the accumulation of *F. alocis* with *P. gingivalis* (Fig. 7A and B). This effect was more pronounced with strain ATCC 35896 than with D-62D. Complementation of the Δ*mfa1* mutation with the wild type allele in trans reduced heterotypic community, in many instances to levels below those of the wild type (Fig. 7B), presumably the result of elevated expression of Mfa1 from the multicopy plasmid. These results indicate that Mfa1 may have a suppressive role in the development of *P. gingivalis*-*F. alocis* communities.

**Figure 8:**
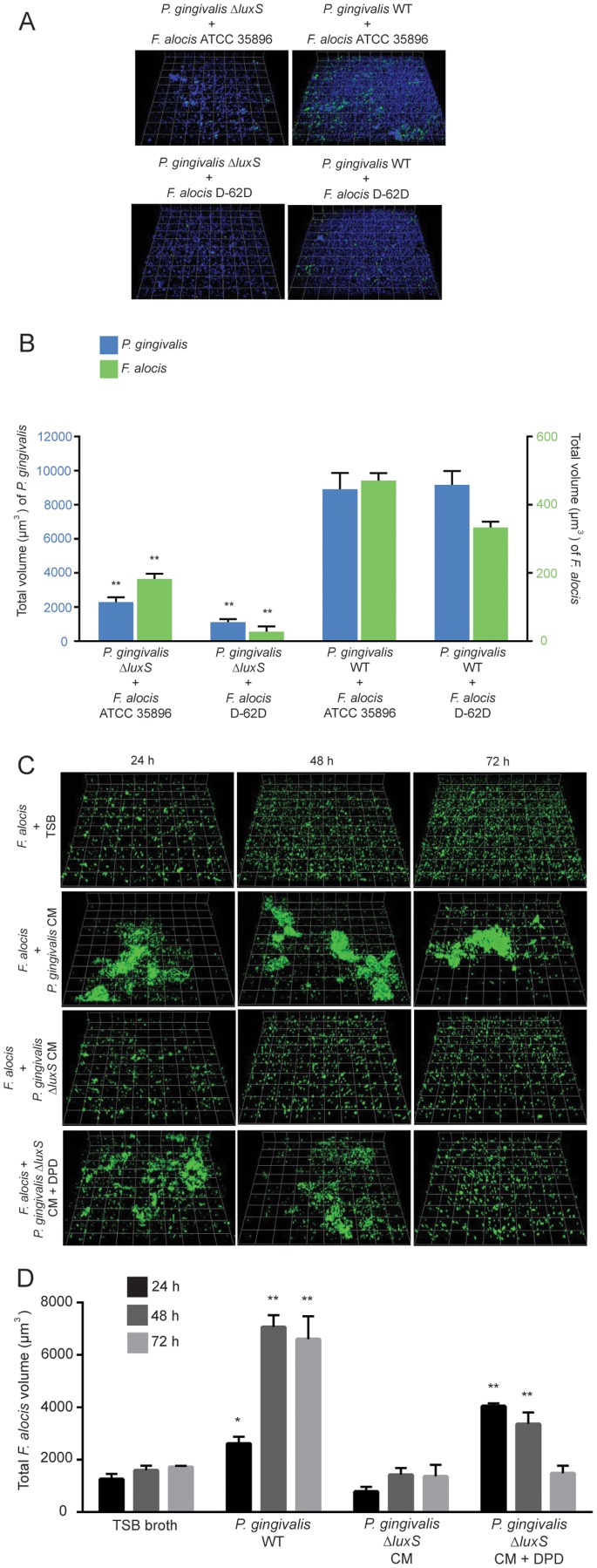
Role of *P. gingivalis* LuxS in dual-species community formation with *F. alocis*. A. *P. gingivalis* ATCC 33277 (WT), and Δ*luxS* (1 × 10^8^, blue, stained with DAPI) were cultured on glass coverslips. *F. alocis* strains ATCC 35896 and D-62D were stained with FITC (green) and reacted with the *P. gingivalis* strains for 72 h. B. Biovolume of *P. gingivalis* or *F. alocis* in dual species communities at 72 h. Data are representative of four independent replicates. P-value compared with control single species communities was calculated by t-test, and significant differences are p<0.01(**). C. Accumulation of *F. alocis* ATCC 35896 stained with FITC (green) and cultured in TSB, conditioned medium (CM) from *P. gingivalis* WT, CM from *P. gingivalis* Δ*luxS*, or CM from *P. gingivalis* Δ*luxS* with 4 μM DPD. D. Biovolume of *F. alocis* ATCC 35896 cultured in TSB, conditioned medium (CM) from *P. gingivalis* WT, CM from *P. gingivalis* Δ*luxS*, or CM from *P. gingivalis* Δ*luxS* with 4 μM DPD. Data are representative of four independent replicates. P-value compared with control single species communities was calculated by t-test, and significant differences are at p<0.05 (*) or p<0.01(**).

To test for possible chemical communication between *P. gingivalis* and *F. alocis* we examined heterotypic community development between *F. alocis* and a mutant of *P. gingivalis* with a deletion in *luxS*, the gene encoding the enzyme responsible for the synthesis of the AI-2 family of signaling molecules. Community biovolume of both *F. alocis* strains was significantly reduced with *P. gingivalis* Δ*luxS* compared to the parental strain (Fig. 8A and B), suggestive of a role for AI-2 in the initial interaction between *P. gingivalis* and *F. alocis*. Interestingly, levels of *P. gingivalis* Δ*luxS* were also reduced in the dual species communities in comparison to the parental strain. Thus, LuxS appears to be required for maximal accumulation of *P. gingivalis* with *F. alocis*, similar to the situation with *P. gingivalis* and *S. gordonii* [26]. To further explore a role for AI-2, we compared *P. gingivalis* Δ*luxS*-*F. alocis* community development in conditioned medium from *P. gingivalis* parental and Δ*luxS* strains. Conditioned medium from the parental, but not the LuxS mutant, strain significantly increased the biovolume of *F. alocis* ATCC 35896 in a community with *P. gingivalis* (Fig. 8C and D). Similar results were obtained with strain D-62D (not shown). Moreover, supplementation of the conditioned medium from the LuxS mutant with DPD, a chemical precursor of AI-2, restored community development to wild type levels for up to 48 h. The effect was lost at 72 h, presumably as a result DPD exhaustion. Quantitative colocalization analysis of *P. gingivalis ΔluxS* and *F. alocis* heterotypic communities showed a decrease in colocalization compared to parental levels (Fig. 6). Collectively, these results show a requirement for interspecies AI-2-dependent signaling for initial association between *F. alocis* and *P. gingivalis*.

### Comparative Effects of *S. gordonii* or *F. nucleatum* on Community Development with *F. alocis*

**Figure 9:**
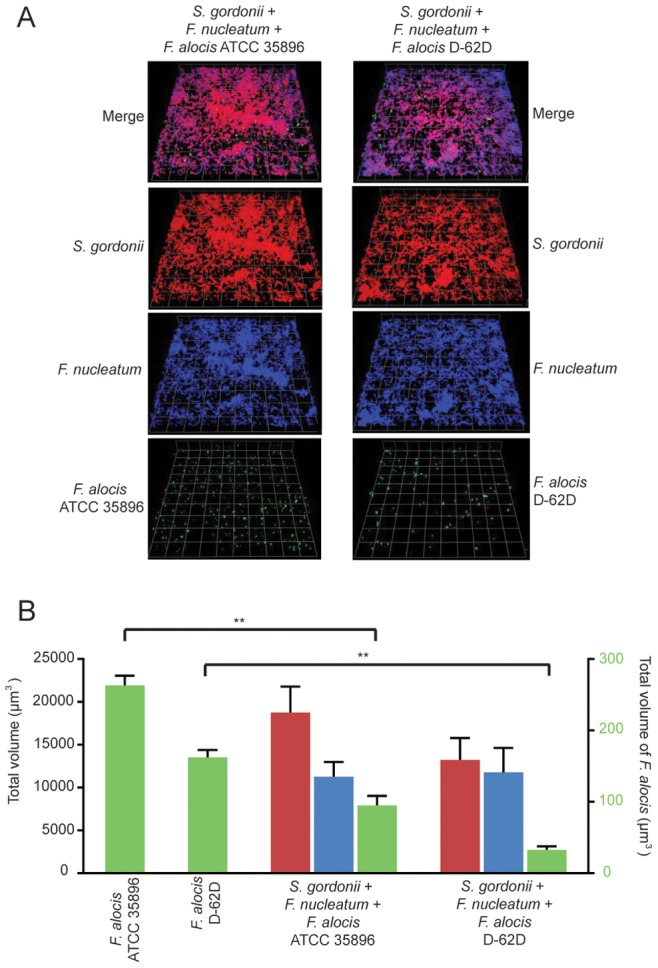
Three-species community formation with *F. alocis*, *S. gordonii* and *F. nucleatum* analyzed by CLSM. A. *S. gordonii* DL1 (red, stained with hexidium iodide), *F. nucleatum* (blue, stained with DAPI) were co-cultured on glass coverslips. *F. alocis* strains ATCC 35896 and D-62D were stained with FITC (green) and reacted with *S. gordonii and F. nucleatum* for 72 h. B. Biovolume of *F. alocis* ATCC 35896 and D-62D, *S. gordonii* DL1 and *F. nucleatum* ATCC 25586 in three species communities. Data are representative of four independent replicates. P-value compared with control single species communities was calculated by t-test, and significant differences are p<0.01(**).

In the mixed species biofilms of the oral cavity *F. alocis* will likely contemporaneously encounter organisms that are synergistic (such as *F. nucleatum*) or are antagonistic (such as *S. gordonii*). To assess the relative contributions of *S. gordonii* and *F. nucleatum,* we generated a three species community comprised of *S. gordonii*, *F. nucleatum* and *F. alocis* (Fig. 9). Accumulation of *F. alocis* in this three-species community was minimal, suggesting that the antagonistic effect of *S. gordonii* supersedes the synergistic effect of *F. nucleatum*. The nature of the synergistic effect is unknown; however, it may not depend on a reduction in pH by *S. gordonii*, as *F. alocis* was capable of monospecies biofilm formation over a pH range of 5–7 (not shown).

## Discussion

Dental plaque is a complex multispecies community that develops temporally and spatially through interbacterial binding and communication systems [27], [28]. Mitis group streptococci such as *S. gordonii* rapidly and avidly attached to saliva-coated tooth surfaces, and these organisms then provide an attachment substratum for later colonizers [3]. Moreover, mitis group streptococci influence the pathogenic potential of later colonizers, a property that has led them to be designated as accessory pathogens in the oral cavity [29]. *F. nucleatum* is abundant in dental plaque and can provide physiological support for other bacteria including *P. gingivalis,* as well as stabilize interbacterial coadhesion networks [30], [31]. Organisms such as *P. gingivalis* and *A. actinomycetemcomitans* are associated with periodontal disease, albeit in the context of raising the pathogenic potential of the microbial community as a whole [1], [32]. Recent research has implicated *F. alocis* as an oral pathogen [17], [19]; however, the colonization mechanisms of *F. alocis* have yet to be studied in detail.

In the present study, the community interactions of *F. alocis* were investigated. *S. gordonii* had a strongly antagonistic effect on *F. alocis,* and colocalization and accretion of *F. alocis* were low in a community with *S. gordonii*. These results suggest that streptococcal rich regions of plaque will be resistant to colonization by *F. alocis*. This is in marked contrast to the interaction between *S. gordonii* and *P. gingivalis*, in which *S. gordonii* provides adhesive and metabolic support for *P. gingivalis* [23], [29], and communities of *S. gordonii* and *P. gingivalis* are more virulent in mouse alveolar bone loss models than either organism alone [33]. Conversely, arginine deiminase produced by *S. cristatus* suppresses fimbrial production by *P. gingivalis* and impedes colonization of the oral cavity [34], [35]. Interbacterial interactions in the oral microbial communities would thus appear to exhibit a high degree of species specificity. Furthermore, while *F. nucleatum* and *F. alocis* were synergistic in accumulation into dual species communities, the antagonistic influence of *S. gordonii* predominated in a three species community. The antagonistic effect of *S. gordonii* would appear, therefore, to extend beyond failure of *S. gordonii* to provide coadhesive support to *F. alocis*. Similarly, host responses to *S. gordonii*-*P. gingivalis* heterotypic communities can show a bias toward *S. gordonii* specific responses. Infection of gingival epithelial cells with *S. gordonii* and *P. gingivalis* together resulted in *S. gordonii* modulating the expression of host genes with a broad diversity of physiological functions, and antagonizing the effect of *P. gingivalis* at the cellular level [36]. Given that oral streptococci can interact with a wide range of bacteria and yeast [29], [37], it is likely that their accessory pathogen role has a major influence on community development and oral health status.

The Mfa1 protein is the structural subunit of the minor fimbriae of *P. gingivalis*. Mfa1 itself can mediate attachment to the streptococcal SspA/B protein [22], [38] and human monocyte-derived dendritic cells [39]. However, Mfa1 is thought to impede the process of internalization into epithelial cells, and the Δ*mfa1* mutant invades epithelial cells more efficiently than the parental strain [40]. Similarly, the presence of the Mfa1 protein is detrimental to community formation with *F. alocis*. Interestingly, initial association between *F. alocis* and *P. gingivalis* was not affected by the loss of Mfa1, rather the accumulation into microcolonies was reduced, indicating that Mfa1 may be involved in the transmission of antagonistic signals between the two organisms. This effect was most pronounced with the type strain, suggestive of heterogeneity of *F. alocis* responses to *P. gingivalis* signals.

The LuxS enzyme is an AI-2 synthase which is responsible for the production of the AI-2 family of inter-convertible signaling molecules. AI-2 is required for optimal accumulation of *P. gingivalis*-*S. gordonii* communities [26], and also controls mixed biofilm formation by various oral streptococcal species [41] and by *Actinomyces oris* and *S. oralis* [42]. In the current study LuxS activity was necessary for maximal association between *P. gingivalis* and *F. alocis*. The LuxS enzyme is also a component of the activated methyl cycle (AMC) [43] and is responsible for recycling of *S*-adenosylhomocysteine (SAH) to homocysteine. Disruption of *luxS* will therefore lead to both a defect in AI-2 mediated signaling and a potential build up of the toxic AMC intermediate, SAH, either of which could affect *P. gingivalis*-*F. alocis* interactions. To distinguish between these possibilities, communities comprised of *P. gingivalis* Δ*luxS* and were chemically complemented with either conditioned medium from the *P. gingivalis* parental strain or with 4,5-dihydroxy-2,3-pentanedione (DPD) an AI-2 precursor. In both cases the wild type phenotype was restored, indicating that the effect of LuxS on *P. gingivalis*-*F. alocis* communities relates to its role in AI-2 signaling. An in silico examination of the currently available *F. alocis* genomic database did not reveal any obvious *luxS* homologs in *F. alocis* and thus *F. alocis* may not produce AI-2 but may be able to sense and respond to the signal, although further studies to resolve this issue are necessary.

*F. alocis* is one of only a few organisms that is associated with both generalized and localized aggressive periodontitis (LAP). The consensus pathogen in LAP is *A. actinomycetemcomitans*, and the type strain of *F. alocis* displayed mutualistic community growth with *A. actinomycetemcomitans*. This result is consonant with the recent report that the presence of a consortium of *A. actinomycetemcomitans*, *S. parasanguinis*, and *F. alocis* is indicative of future bone loss in LAP [44]. Interestingly, the more recent clinical isolate D-62D did not show this synergy with *A. actinomycetemcomitans*. This result, along with other differences between the type strain and D-62D reveals heterogeneity within the *F. alocis* taxon, particularly with regard to potential involvement in LAP. As few *F. alocis* isolates have been studied, the existence of subgroups with differing properties are yet to be defined. However, in a proteomic study of *F. alocis* strains, Aruni et al. [19] found more cell wall anchoring proteins in D-62D compared to ATCC 35896, which may have relevance for interactions with *A. actinomycetemcomitans*. Future studies involving additional *F. alocis* strains will be necessary to more fully delineate the interspecies coadhesion profile of the organism. Isolates of *A. actinomycetemcomitans* from the oral cavity also display heterogeneity with respect to levels of leukotoxin and fimbrial production [45], [46], and different strains of *A. actinomycetemcomitans* therefore could also exhibit different patterns of reactivity with *F. alocis*.

## Conclusions

While the dental plaque biofilm develops on all subgingival tooth surfaces in the oral cavity, periodontal disease is more usually localized to specific sites. Thus, spatial variations in the pathogenic potential of the biofilm communities exist. Complex synergistic and antagonistic interactions occur within oral microbial communities and these underlie the success or failure of microbial colonization. The results of this work indicate that the pattern of colonization of *F. alocis* depends heavily on the antecedent inhabitants of the microbial community. Although dental biofilms can comprise several hundred bacterial species, by practical necessity *in vitro* studies such as these are limited in the number of organisms and strains that can be investigated, and we recognize that the presence of other bacterial species could modulate the interactions reported herein. Nonetheless, the network of interactions established for *F. alocis* provides mechanistic insights into the colonization strategies of the organism and form a framework for future studies to define the molecular basis of *F. alocis* colonization and community formation.
